# Tabes Mesenterica, with Cases

**Published:** 1870-05

**Authors:** F. K. Bailey

**Affiliations:** Knoxville, Tenn.


ARTICLE XV.

### TABES MESENTERICA, WITH CASES.

BY F. K. BAILEY, M.D., Knoxville, Tenn.

*Nov. 9th, 1867.* An Irish woman brought her child (male), about one year old, to my office, when I found its history as follows:—At times during the previous summer it had diarrhœa, attended with vomiting, which was without doubt true cholera infantum. Emaciation was extreme, and the skin hung in folds upon the face and limbs. The eyes were sunken, the surface of a bluish hue, and the expression extremely haggard. The abdomen was enormously enlarged, with a knotty feel to the surface. There was a hardness over the liver, and dulness in the whole right side of the abdomen.

The stools were frequent, milky, and very offensive. Diagnosis—enlargement of the mesenteric glands, with engorgement of the liver. Prognosis very unfavorable.

Prescribed as follows:—

℞. Liquor Iod. Ferri, ------------------ ʒij.

Syr. Sarsa. Compos.,---------------- ℥ss.

Sulph. Cinchoniæ,------------------ grs. viij.

Syr. Simplices, ---------------------- ℥j.

Aquas Cinnamomi, -------------------- ℥ss.

M. Liq. Two-third teaspoonful four times daily. Diet—beef soup and milk.

*16th.* The woman called, when I found the child some better, but the stools rather more frequent and copious. Directed a continuation of the mixture, and for the diarrhœa, as follows:

℞. Syr. Rhei Aromat., ---------------------- ℥j.

Tr. Kino, -------------------------------- ʒij.

Tr. Opii Camph., -------------------------- ʒiij.

M. Liq. 30 drops two or three times daily.

*Nov. 28th.* Again called. Marked improvement. Less distension of the abdomen and some gain in flesh. The enlargement in the hepatic region still obvious. Directed the following:

℞. Liq. Iod. Ferri, ---------------------- ʒiij.

Sul. Cinchoniæ, ---------------------- grs. viij.

Iod. Potassii, ------------------------ ℈ij.

Syr. Simp., -------------------------- ℥iss.

Aquæ Puræ, -------------------------- ℥ss.

M. Liq. Two-third teaspoonful three times daily, externally, to the hepatic region.

℞. Iod. Potassii, ------------------------ ʒss.

Tr. Opii, -------------------------------- ʒij.

Oleii Olivæ, ---------------------------- ℥j.

From this time the child improved slowly. The distension subsided very much, but the knotty feel was perceptible for some months.

In September, 1868, I was called to see the child, and found it suffering from diarrhœa and vomiting, considerably emaciated, and with tumidity of the abdomen, but no knottiness over the mesentery. Gave iod. iron, with sul. quinine; pulv. Doveri at night, and improvement was immediate. Saw no more of the child till June 10th, 1869, when I was called, and found it laboring under cholera infantum. Although the child had grown somewhat since last fall, still he was small and weak, and could walk with difficulty, Tonic alteratives and opiates soon checked the excessive action of the bowels, and in a few day after, no diarrhœa remained.

*July 22d.* Another relapse of diarrhœa, with extreme exhaustion. There was abdominal fulness, or evident glandular enlargement. Prescribed iod. iron, with sul. quinine, and opiates, *pro re nata*, and convalescence was soon apparent.

*January 1st, 1870.* There has been no return of diarrhœa since last summer, and the little fellow is becoming a fat, stout boy, and able to do justice to a square meal, of an Irish mother's cooking.

*March 8th, 1869.* Called to see a little girl three years old, child of unmixed African parents. Found the little creature sitting upon a low chair, a perfect picture of abject wretchedness. She had been sick a long time from diarrhœa, which nothing had seemed to control in the least. The face and head looked like a skeleton upon which some black leather had been stretched. The voice was feeble and sepulchral. The abdomen was enormously distended, and the lower limbs looked, as they hung from the edge of the chair, like two pieces of tarred rope dangling from the lower side of a blackened sphere.

I could give no encouragement in the way of cure, but prescribed iod. iron, with iod. potassii and sulph. cinchoniæ. I saw the child occasionally during the month, and, on the 30th, saw there was some gain in strength, both in limb and voice. During the summer, the little patient was kept upon iron and the preparations of cinchoniæ. The diarrhœa was difficult of control; but nothing, except tonics, was of any avail.

Some time in August, the child died. A week or more before death, the distension subsided in the abdomen, when there was brought to view and feeling a large lump, or cluster, of a nodulated character, in the front parietes, which, doubtless, were the enlarged mesenteric glands. There was no *post mortem* examination.

*July 18th, 1869.* Called to see a child, 18 months old, of African parents, which had had summer complaint for some weeks. The abdomen was but slightly distended, and the lumpy feel was very apparent. Discharges, milky and frequent; great thirst, and very rapid circulation. Prescribed as follows:—

℞. Liquor Iod. Iron, ---------------------- ℨij.

Iod. Potassii,---------------------------- ℨss.

Sulph. Quinine, ------------------------ grs. x.

Syr. Simp., ----------------------------- ℥iij.

M. Liq. Three-fourth teaspoonful four times daily. In a few weeks, this child was well; but occasional relapses of diarrhœa occurred.

Besides the above, I have seen two or three cases in which ordinary summer complaint was accompanied by an incipient enlargement of the mesentery; but by appropriate and prompt tonic alterative medication, the progress was suspended.

I do not think that there was any strumous diathesis in either parents of the children, whose cases are above mentioned.

Since writing the above, I saw the first mentioned patient, while incidentally in the neighborhood.

Growth has been slow, and the abdomen is considerably distended. The fulness is probably a result of deficient tone in the muscular walls of the intestines, and not from glandular enlargement; because nutrition seems to be good, and the child has a good share of adipose and muscle.

*March 22d, 1870.*

